# The Niemann–Pick C1 Protein of Patients with Hepatocellular Carcinoma Is Associated with Survival Time in Males and Tumor Size in Females

**DOI:** 10.3390/biomedicines13071707

**Published:** 2025-07-13

**Authors:** Florian Weber, Katja Evert, Alexander Scheiter, Sophie von Sachsen-Coburg, Kirsten Utpatel, Christa Buechler

**Affiliations:** 1 Institute of Pathology, University of Regensburg, 93053 Regensburg, Germany; florian.weber@klinik.uni-regensburg.de (F.W.); katja.evert@klinik.uni-regensburg.de (K.E.); alexander.scheiter@ukr.de (A.S.); kirsten.utpatel@klinik.uni-regensburg.de (K.U.); 2 Department of Internal Medicine I, Regensburg University Hospital, 93053 Regensburg, Germany

**Keywords:** disease etiology, sex, UICC score, survival

## Abstract

**Background/Objectives:** The Niemann–Pick C1 (NPC1) protein regulates cellular cholesterol homeostasis, which is disrupted in hepatocellular carcinoma (HCC). Sex differences in cholesterol metabolism may also be related to NPC1 expression in HCC. A sex-specific analysis was, therefore, performed to investigate this further. **Methods:** The expression of NPC1 protein in hepatocytes was assessed using immunohistochemistry in HCC tissues from 264 male and 59 female patients, as well as in non-tumor tissues from 41 males and 7 females. **Results:** The disease etiology was documented for 40% of these patients, and NPC1 protein levels in the tumors of patients with alcoholic, metabolic, and viral liver disease were comparable. The severity of underlying liver fibrosis was similar in both females and males. No difference in hepatocyte NPC1 protein expression was observed between males and females in non-tumor and tumor tissues. However, NPC1 expression was strongly increased in tumor tissues in both sexes. NPC1 protein levels were positively associated with T stage and Union for International Cancer Control (UICC) stage in both sexes. NPC1 protein levels were negatively correlated with overall survival, recurrence-free survival, and metastasis-free survival time in males only. Univariate Cox regression analysis showed a significant association of NPC1 protein levels with metastasis-free survival in males. Positive correlations of NPC1 protein with tumor size and negative associations with tumor inflammation were observed only in women. **Conclusions:** This study showed that hepatocyte NPC1 protein levels are highly elevated in HCC tissue in both sexes but are more closely associated with survival in male patients than in female patients.

## 1. Introduction

Hepatocellular carcinoma (HCC) is the most common primary liver tumor [1]. HCC arises in the chronically injured liver, most frequently caused by viral infections, alcohol abuse, and metabolic dysfunction-associated steatotic liver disease (MASLD), the latter of which has become the leading cause of HCC in the Western world [2,3]. Liver inflammation triggers fibrosis, which can progress to cirrhosis and HCC. MASLD-HCC can also occur in the non-cirrhotic liver, but the incidence of HCC is much higher in the cirrhotic liver [2,3]. The treatment of HCC poses significant challenges, particularly a high recurrence rate and limited survival, with current clinical therapies often falling short [4,5,6]. This highlights the urgent need for comprehensive research into the molecular mechanisms underlying HCC progression.

HCC tissues show increased cholesterol biosynthesis, with sterol regulatory element binding protein 2, the major transcription factor regulating cholesterol biosynthesis, activated in HCC cells [7]. Niemann–Pick C1 (NPC1) protein plays a central role in cellular cholesterol transport and is also upregulated in HCC tissues [8,9]. NPC1 protein localizes to endosomal and lysosomal membranes and mediates the export of cholesterol from these organelles, a pathway essential for cellular cholesterol homeostasis and cell function [10]. High NPC1 protein in Chinese hamster ovary cells elevated cellular cholesterol levels and increased cholesterol biosynthesis [11], suggesting that NPC1 plays a crucial role in cell proliferation, which is essential for carcinogenesis. Overexpression of NPC1 in human highly metastatic liver cancer cells (MHCC)-97H and Huh7 cells increased proliferation and cell viability. In addition, NPC1 knockdown reduced colony formation, migration, and invasion of the cells, suggesting a function of NPC1 in tumor development and metastasis [12]. Huh7 and HepG2 cell proliferation were also impaired by NPC1 knockdown [13]. Knockdown of NPC1 in the murine Hepa1–6 cells did not affect cell proliferation [9]. This study showed that NPC1 promotes liver tumor progression by enhancing the recruitment of neutrophils to the tumor environment [9]. NPC1 can also stabilize transforming growth factor (TGF)-β receptor type-1, thereby increasing the tumor-promoting activities of TGF-β [8]. TGF-β has been described as inducing cell growth arrest and acting as a tumor suppressor in early HCC. In the late stages of HCC, TGF-β contributes to HCC progression through various mechanisms [14].

*NPC1* mRNA and NPC1 protein are highly elevated in HCC tissues, and NPC1 protein in tumors is associated with poor prognosis [8,13]. *NPC1* mRNA expression increased with higher tumor stages in HCC and was further increased in patients with metastasis [9]. Patients with lower *NPC1* mRNA levels had better overall and relapse-free survival [9]. 

Higher prevalences of chronic liver diseases and HCC in males compared to females are well known. In Germany, there is a 2.4-fold higher prevalence of HCC in males than in females [15]. These sex-specific differences result from a complex mix of factors, including gender-specific lifestyle, hormonal differences, and genetic variations [16]. Whether women have a better outcome from HCC is less clear. In a study of approximately 1500 patients with HCC, the overall survival of both sexes was similar [17]. Another study reported better outcomes in women, with earlier detection of HCC and a better response to initial therapy [18]. In Germany, the 5-year survival rate of males is 18%, which is higher than that of females, with a 14% survival rate [15].

Sex-specific effects of NPC1 loss have been described, and liver weight and hepatic caveolin-1 levels are differentially affected in male and female mutant mice [19,20]. To our knowledge, sex-specific analysis of NPC1 protein levels and associations with HCC severity has not been performed. Cholesterol metabolism differs in males and females [21], and was also shown to contribute to sex-specific disparities in liver cancer [22,23]; however, whether this translates to NPC1 expression is unknown. Sex-specific studies are important for understanding the higher incidence of HCC in males [15]. Cholesterol-lowering drugs have been shown to protect against HCC [24] and may emerge as adjuvants for HCC immunotherapies [25]. However, immunotherapies for HCC appear to be less effective in females [26], and sex-specific studies are required to enhance the response to HCC treatment in both sexes. Nevertheless, such studies are comparatively rare [16]. Interestingly, simvastatin was found to upregulate the NPC1 protein in macrophage foam cells [27], but it is unclear whether statins also affect hepatocyte NPC1 protein levels in HCC. Sex-based disparities in lipid-lowering therapy and statin intolerance must also be considered here [28].

This study used immunohistochemistry to determine the NPC1 protein levels in non-tumor and HCC tissues of females and males to identify sex-specific associations between the NPC1 protein and the severity of the underlying liver disease, tumor size, tumor stage, and survival in a large cohort of German patients with HCC.

## 2. Materials and Methods

### 2.1. Patients

Between 2000 and 2021, HCC tissue was collected from 323 patients—264 men and 59 women. The majority of patients came from the eastern region of Bavaria. Non-tumor tissues from 48 HCC patients (7 women and 41 men) were also included.

Experienced pathologists selected representative areas and evaluated haematoxylin and eosin-stained sections of HCC tissue. Using standard procedures described previously [29], seven tissue microarrays (TMAs) were generated with up to 60 tissue cores per TMA. The final TMA contained one core from each tumor. Pathological primary tumor extent (pT stage) and disease stage were determined using the TNM classification system according to the 8th edition of the Union for International Cancer Control (UICC) staging system [30,31].

Serum alpha-fetoprotein was analyzed as a routine parameter in the Institute of Clinical Chemistry and Laboratory Medicine, University Hospital Regensburg.

### 2.2. Immunohistochemistry

The NPC1 antibody (order number: E7S4N) was obtained from Cell Signaling Technology Europe B.V. (Leiden, The Netherlands). Sections of the TMA blocks, 4 μm thick, were deparaffinized and treated with Tris-EDTA buffer (pH 9) for 5 min at 120 °C for immunohistochemistry. Endogenous peroxidase was blocked using a peroxidase blocking solution (Dako, Glostrup, Denmark), and the antibody (1:50 dilution) was incubated for 30 min at room temperature. The Dako EnVision™ Detection System, Peroxidase/DAB, and anti-mouse/anti-rabbit antibodies (Dako, Glostrup, Denmark) were used for staining. The slides were then counterstained with haematoxylin.

The H-score was used for quantification by an experienced pathologist (F.W.), calculated as the percentage of positive cells multiplied by intensity from 0 to 3. The score ranges from 0 (completely negative) to a maximum of 300 (all strongly positive).

### 2.3. Histological Scores

The histological evaluation of the degree of inflammation in HCC tissue and liver fibrosis was performed by experienced liver pathologists K.U. and K.E. A 4-point scale was used to score intratumoral inflammation, with 0 representing no inflammation and 3 representing severe inflammation. Liver parenchymal fibrosis in the surgical specimens was graded using the Ishak fibrosis score, which is a seven-point grading system where 0 indicates no fibrosis and 6 indicates cirrhosis [32].

### 2.4. Statistics

The median, minimum, and maximum values are given in the tables. In the diagrams, data are shown as boxplots, which show the median values, the 25th and 75th quantiles, and the minimum and maximum data values. Outliers are plotted as individual circles.

The NPC1 protein levels in HCC tissues were not normally distributed, which was determined using the Shapiro–Wilk and the Kolmogorov–Smirnov statistics (*p* < 0.001 for the Shapiro–Wilk and the Kolmogorov–Smirnov statistics), and non-parametric statistical tests were used. These tests were the Mann–Whitney U-Test for comparison of two groups and the Kruskal–Wallis test with post hoc Bonferroni correction for comparison of more than two groups. Univariate Cox regression and Receiver operating characteristic curves were also calculated (SPSS Statistics 26.0, IBM, Leibniz Computing Centre, Munich, Germany). A *p*-value of less than 0.05 was considered significant.

## 3. Results

### 3.1. NPC1 Protein Expression in HCC Tissues of the Entire Cohort

**Figure 1:**
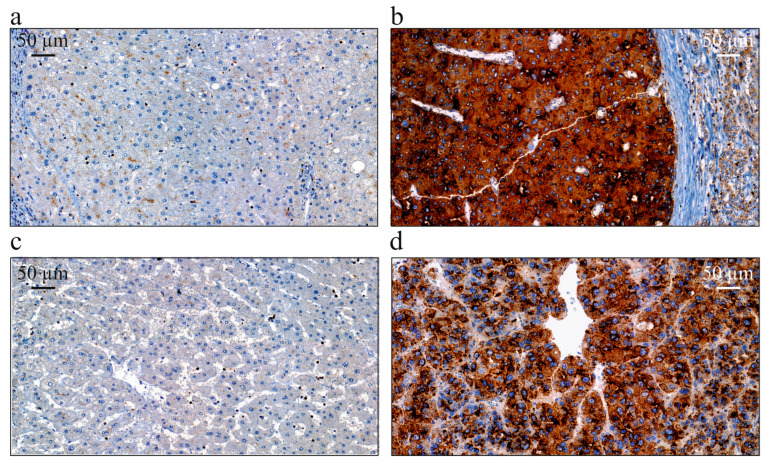
Immunohistochemistry of NPC1 protein in non-tumor and tumor tissues of randomly selected patients with HCC. (**a**) NPC1 protein in the non-tumor tissue of a female patient (age: 51 years, tumor size: 2.6 cm, UICC score: 1, fibrosis grade: 6; HCV infection); (**b**) NPC1 protein in the HCC tissue of a female patient (age: 50 years, tumor size: 9.5 cm, UICC score: 2, fibrosis grade: 6; HCV infection); (**c**) NPC1 protein in the non-tumor tissue of a male patient (age: 57 years, tumor size: 4.2 cm, UICC score: 1, fibrosis grade: 6; alcoholic etiology); (**d**) NPC1 protein in the HCC tissue of a male patient (age: 46 years, tumor size: 16.5 cm, UICC score: 4, fibrosis grade: 0; unknown etiology) (400-fold magnification in all images; scale bar: 50 µm).

NPC1 protein levels in non-tumor and HCC tissues were determined by immunohistochemistry (Figure 1), and the expression in hepatocytes was scored.

**Figure 2:**
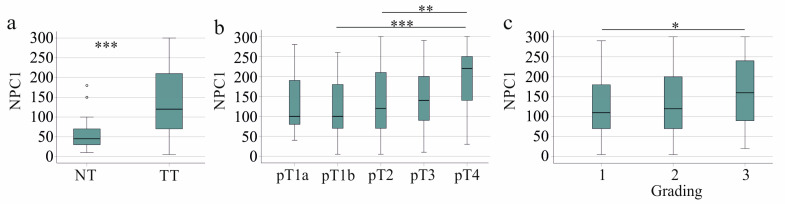
NPC1 protein in non-tumor (NT) and tumor tissues (TT) of patients with HCC and its association with disease severity in the entire cohort. (**a**) NPC1 protein levels in NT of 48 patients with HCC and TT of 323 patients; (**b**) NPC1 in the HCC tissues according to T-stages; (**c**) NPC1 in the HCC tissues according to grading. * *p* < 0.05, ** *p* < 0.01, *** *p* < 0.001.

NPC1 protein was significantly more expressed in the tumors than in the non-tumor tissues (Figure 2a). NPC1 protein was positively related to tumor stage and grading (Figure 2b,c). Patients with and without lymph node invasion had similar hepatic NPC1 protein levels (*p* = 0.655). NPC1 protein in the HCC tissues positively correlated with the UICC score (r = 0.196, *p* < 0.001) but not with tumor size (r = 0.092, *p* = 0.100). Moreover, NPC1 protein in the HCC tissues positively correlated with serum alpha-fetoprotein (AFP), which had been documented for 175 patients (r = 0.342, *p* < 0.001).

Age was unrelated to hepatic NPC1 protein levels (r = 0.015, *p* = 0.795). Inflammation of the tumors, as scored on an arbitrary 4-tiered scale, was negatively correlated with liver NPC1 protein levels (r = −0.130, *p* = 0.023). However, no associations were observed with the grade of liver fibrosis (r = −0.073, *p* = 0.215).

Overall survival time (r = −0.162, *p* = 0.007), metastasis-free survival time (r = −0.194, *p* = 0.001), and recurrence-free survival time (r = −0.165, *p* = 0.006) negatively correlated with NPC1 protein levels in the HCC tissues. The area under the receiver operating characteristics curve (AUROC) for discriminating between 5-year overall survivors and non-survivors was 0.531 (±0.035; *p* = 0.373), for 5-year metastasis-free survivors and non-survivors was 0.550 ± 0.035 (*p* = 0.165), and for discriminating between recurrence-free survivors and non-survivors was 0.560 ± 0.037 (*p* = 0.113).

### 3.2. NPC1 Protein Expression and Etiology of Underlying Liver Disease

**Figure 3:**
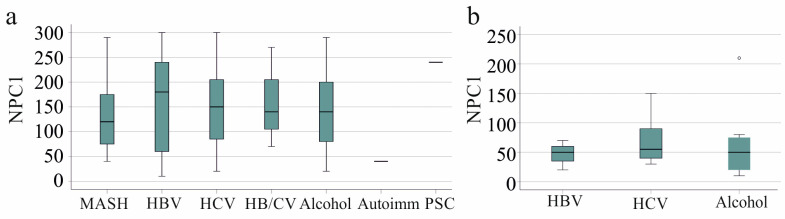
NPC1 protein in non-tumor and tumor tissues of patients with HCC and its association with the underlying disease etiology of liver disease in the entire cohort. (**a**) NPC1 protein levels in the HCC tissues of patients with different disease etiologies. Kruskal–Wallis test with post hoc Bonferroni correction revealed no significant differences between these patients; (**b**) NPC1 protein levels in the non-HCC tissues of patients with different disease etiologies. Kruskal–Wallis test with post hoc Bonferroni correction revealed no significant differences between these patients. Autoimm: autoimmune; HBV: hepatitis B virus, HCV: hepatitis C virus, HB/CV: HBV and HCV infection, MASH: metabolic dysfunction-associated steatohepatitis, PSC: primary sclerosing cholangitis. The small circle in Figure 3b is an outlier.

Of the patients from whom HCC tissues were obtained, the underlying liver disease was metabolic dysfunction-associated steatohepatitis (MASH) in 16 patients; hepatitis B virus (HBV) in 21 patients; hepatitis C virus (HCV) in 39 patients; HBV and HCV infection in 3 patients; alcohol-related liver disease in 45 patients; and autoimmune hepatitis and primary sclerosing cholangitis in one patient each. The underlying disease of 197 patients was not defined by the clinicians. NPC1 protein expression in HCC tissues from patients with different disease etiologies was similar (Figure 3a).

In the cohort of patients from whom non-HCC tissues were obtained, the underlying liver disease was HBV in three patients, HCV in ten patients, and alcohol in eight patients, with similar NPC1 protein levels observed between these groups (Figure 3b). The etiology of 27 patients was unclear.

### 3.3. Male and Female Patients

It should be noted that non-tumor tissues of only seven females were included, and this limits the statistical power. Men and women in the patient cohorts from which non-tumor and tumor tissue were obtained were similar in age, T stage, and tumor grade, lymph node invasion, grading, and UICC scores (Table 1). There was no difference in the T stage, tumor grade, lymph node invasion, grading, and UICC scores between patients where tumor tissue had been obtained and those where non-tumor tissue had been obtained, showing similar disease severities in these cohorts. This applied to both male and female patients.

The tumor size of females was larger, and this was significant in the cohort where tumor tissues were obtained (Table 1). Overall survival, metastasis-free, and recurrence-free survival times for both sexes were similar (Table 1). Tumor inflammation scores and fibrosis scores did not significantly differ between males and females. NPC1 protein levels of both sexes were similar (Table 1).

Of the patients from whom HCC tissue was obtained, the underlying liver disease was MASH in 2 female and 14 male patients, HBV in 2 female and 19 male patients, HCV in 10 female and 29 male patients, and HBV/HCV coinfection in 1 female and 2 male patients, and alcohol in 2 females and 43 males. Autoimmune hepatitis and PSC were only found in female patients. The disease etiology differed significantly between female and male patients (*p* = 0.007).

In the cohort of patients from whom non-HCC tissues were obtained, the underlying liver disease was HBV in one female and two male patients, HCV in two female and eight male patients, and alcohol in eight male patients. There was no difference between the sexes (*p* = 0.460).

In males, NPC1 protein in the non-HCC tissues was not related to T stage, lymph node invasion, grading, tumor size, and UICC scores (*p* > 0.05 for all). Overall, metastasis-free and recurrence-free survival time, as well as hepatic inflammation and fibrosis scores, did not correlate with NPC1 protein levels (*p* > 0.05 for all). Non-tumor tissues of only seven females were included, and correlation analysis was not meaningful.

### 3.4. NPC1 Protein Expression in HCC Tissues of Male Patients

**Figure 4:**
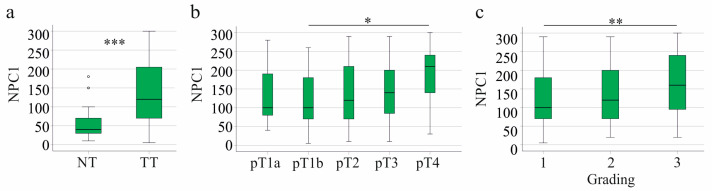
NPC1 in non-tumor (NT) and tumor tissues (TT) of male patients with HCC and its associations with disease severity. (**a**) NPC1 protein in NT and TT of the male patients; (**b**) NPC1 protein in the HCC tissues according to T-stages; (**c**) NPC1 protein in the HCC tissues according to grading. * *p* < 0.05, ** *p* < 0.01, *** *p* < 0.001. The small circles in Figure 4a are outliers.

In male patients, NPC1 protein levels in the tumors were increased compared to non-tumor tissues (Figure 4a).

NPC1 protein was higher in male patients with tumor stage pT4 compared to pT1b (Figure 4b) and was positively related to grading (Figure 4c). Male patients with and without lymph node invasion had similar hepatic NPC1 protein levels (*p* = 0.919). NPC1 protein in the HCC tissues positively correlated with the UICC score (r = 0.170, *p* = 0.006) but not with tumor size (r = 0.035, *p* = 0.578).

In male patients, NPC1 protein levels in HCC tissues were negatively correlated with survival time. Overall survival time (r = −0.160, *p* = 0.016), metastasis-free survival time (r = −0.185, *p* = 0.005), and recurrence-free survival time (r = −0.144, *p* = 0.030) were negatively correlated with hepatic NPC1 protein. The AUROC was 0.526 ± 0.039 for overall survival (*p* = 0.508), 0.548 ± 0.039 for metastasis-free survival (*p* = 0.227), and 0.554 ± 0.042 for recurrence-free survival (*p* = 0.205), showing that the NPC1 protein could not distinguish between 5-year survivors and non-survivors. Univariate Cox regression analysis revealed a significant association between the NPC1 protein and metastasis-free survival (*p* = 0.034), though the associations with recurrence-free and overall survival were not significant (*p* = 0.071 for both).

Age of the male patients was not related to hepatic NPC1 protein levels (r = 0.054, *p* = 0.380). Tumor inflammation (r = −0.094, *p* = 0.135) and fibrosis stages (r = 0.035, *p* = 0.578) did not correlate with NPC1 protein levels.

AFP and NPC1 protein were positively related (r = 0.343, *p* < 0.001). AFP did not correlate with stages of the underlying liver fibrosis, tumor size, and tumor inflammation (*p* > 0.05 for all).

The male patients with different disease etiologies had similar NPC1 protein levels in their tumors (*p* = 0.696).

### 3.5. NPC1 Protein Expression in HCC Tissues of the Female Patients

**Figure 5:**
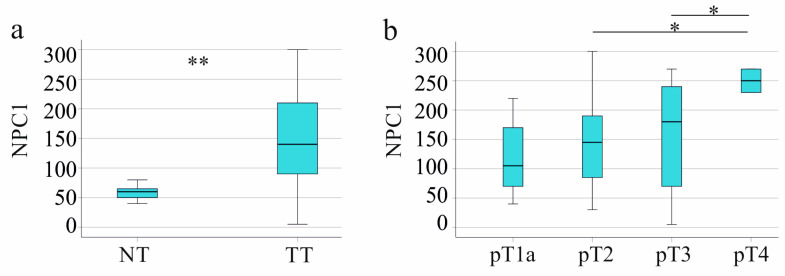
NPC1 protein in non-tumor (NT) and tumor tissues (TT) of female patients with HCC and its associations with disease severity. (**a**) NPC1 protein in NT and TT of the female patients; (**b**) NPC1 protein in the HCC tissues according to T stages. * *p* < 0.05, ** *p* < 0.01.

In female patients, NPC1 protein levels in the tumors were significantly higher compared to non-tumor tissues (Figure 5a). A limitation of this analysis is that the non-tumor tissues of only seven women were included.

NPC1 protein was higher in female patients with tumor stage pT4 compared to pT3 and pT2 (Figure 5b) but was not related to grading (*p* = 0.776). Female patients with and without lymph node invasion had similar hepatic NPC1 protein levels (*p* = 0.308). NPC1 protein in the HCC tissues positively correlated with the UICC score (r = 0.346, *p* = 0.008) and tumor size (r = 0.316, *p* = 0.015).

In female patients, NPC1 protein levels did not correlate with survival time. Overall survival time (r = −0.166, *p* = 0.251), metastasis-free survival time (r = −0.220, *p* = 0.124), and recurrence-free survival time (r = −0.243, *p* = 0.090) were not correlated with the hepatic NPC1 protein. The AUROC was 0.556 ± 0.084 for overall survival (*p* = 0.507), 0.556 ± 0.086 for metastasis-free survival (*p* = 0.510), and 0.590 ± 0.085 for recurrence-free survival (*p* = 0.293). Univariate Cox regression analysis revealed no association between the expression of the NPC1 protein and either metastasis-free survival (*p* = 0.302), recurrence-free survival (*p* = 0.167), or overall survival (*p* = 0.388).These results show that the NPC1 protein levels in the tumors of female patients are not related to survival.

Age of the female patients was not related to hepatic NPC1 protein levels (r = −0.104, *p* = 0.433). Intratumoral inflammation (r = −0.294, *p* = 0.029) negatively correlated with NPC1 protein in the tumor, which was not associated with the fibrosis stages of the underlying liver disease (r = 0.017, *p* = 0.907).

AFP levels were positively correlated with NPC1 protein levels (r = 0.353, *p* = 0.030) and tumor size (r = 0.316, *p* = 0.015) but negatively correlated with tumor inflammation (r = −0.294, *p* = 0.029).

The disease etiology of only 19 females was known, and statistical analysis was not performed.

## 4. Discussion

This study showed that the levels of the NPC1 protein were similarly elevated in tumors from both male and female patients. NPC1 protein levels were positively correlated with tumor size in females and tumor grade in males, and were negatively associated with survival time in males, demonstrating sex-specific associations between tumor NPC1 protein levels and tumor size, tumor cell differentiation, and disease outcome.

Common disease etiologies for cirrhosis in Germany between July 2012 and February 2014 were alcohol (52%), HCV (28%), HBV (14%), and MASH (6%) [33]. For 60% of our patients, the etiology of chronic liver disease was not indicated by the clinicians. MASH is a major cause of cryptogenic liver cirrhosis [34], and most of these undefined patients may have underlying MASH, which is still difficult to diagnose [35].

Of our cases with a known etiology of liver cirrhosis, 35% had alcoholic liver cirrhosis, 31% had HCV, 2% had HBV, and 13% had MASH. The etiology of liver cirrhosis changes over time and also varies according to factors such as population [36], making it difficult to compare our cohort, including samples from 2000 to 2021, with other studies.

The etiology of chronic liver diseases also varies by sex. Alcoholic liver disease and chronic HBV infection were more prevalent in males, in line with previous studies [37]. MASH was diagnosed in 10% of females and 13% of males, suggesting a similar prevalence to that described in studies comparing postmenopausal women and age-matched men [37,38]. The median age of our female patients was 65 years, indicating that most of them were postmenopausal. In accordance with the twofold higher prevalence of HCV in men [37,38], our cohort had an almost 3-fold higher proportion of HCV-positive men. Autoimmune hepatitis, which has a higher prevalence in women, and PSC, which may be more prevalent in men [37,38], were rare and were only diagnosed in female patients.

The incidence of HCC has been found to vary significantly between men and women. A mixture of biological and environmental factors [39,40] and variations in the HCC proteome [40] may account for this discrepancy. HCC is more common in men [41], and there were 5.5 times more men than women in our HCC cohort.

NPC1 protein levels in the tumor and non-tumor tissues were unrelated to the etiology of chronic liver diseases, and were similar in viral infections, alcoholic liver cirrhosis, and MASH [42]. NPC1 protein levels did not correlate with fibrosis grade, showing that their increase in HCC is a feature of this malignancy rather than of the severity of the underlying liver disease.

Consistent with previous studies, NPC1 protein was increased in HCC tissues compared to non-tumor tissues [9,12]. It was similarly increased in the tumor tissue of the male and female patients. NPC1 protein positively correlated with the UICC score and AFP, an observation made in both sexes. NPC1 protein in tumors was positively associated with tumor stages in both male and female patients.

Larger tumor size is associated with poorer survival [43], and the female patients in our cohort had larger tumors. However, overall, metastasis-free and recurrence-free survival were similar between the sexes. Although HCC-specific mortality increased with tumor size in both sexes, females with large tumors had better survival rates than males [44], which is consistent with the current observation.

Positive correlations of NPC1 with tumor size were significant only in female patients. In females, NPC1 in HCC tissues was not associated with survival. This shows that the increase in NPC1 protein levels in females with larger tumor sizes is not related to a worse outcome.

In male patients, tumor NPC1 protein levels were not related to tumor size but were negatively correlated with survival time. Therefore, higher levels of NPC1 protein in HCC tissues in males are associated with poorer outcomes. The association of NPC1 with grading was only significant in males, suggesting that NPC1 protein levels in less differentiated tumors are increased. Poor differentiation of tumors is associated with a worse prognosis [42], and this may partly explain the correlation between NPC1 protein levels and outcome in male patients. It should be noted that the association between NPC1 protein levels and survival was weak. This is contrary to previous studies, which showed that high levels of NPC1 were clearly linked to a worse outcome [8,13].

Accordingly, NPC1 protein levels in tumors did not discriminate between survivors and non-survivors of either sex in our cohort and cannot, therefore, be used as a prognostic biomarker. AFP is specifically increased in patients with HCC, and high levels are prognostic of tumor dedifferentiation and poorer survival [45]. NPC1 in the HCC tissue positively correlated with serum AFP levels in both sexes. High AFP levels were found to be associated with female sex [46] and male sex [47], and there was no difference between the sexes in our cohort. AFP has immunosuppressive activity [45] and is negatively correlated with tumor inflammation in females. In HCC tissues, high levels of AFP and NPC1 are associated with less inflammation in females but not in males. Sex-specific associations with inflammation in experimental HCC have been described [48], and the role of AFP and NPC1 in this context requires further study.

High levels of NPC1 have been shown to increase the proliferation of the human MHCC-97H, Huh7, and HepG2 cells [12,13] but did not affect the proliferation of murine Hepa1–6 cells [9], suggesting species-specific effects of NPC1 overexpression. All of these cell lines are derived from males, and, to our knowledge, whether cells from female patients have increased proliferation upon NPC1 overexpression, consistent with its positive correlation with tumor size in females, has not been investigated.

Itraconazole, an antifungal medication, has been identified as a direct inhibitor of NPC1 [49] and exerts anti-HCC effects [50]. In male adult Sprague Dawley rats, itraconazole given in parallel with diethylnitrosamine reduced inflammation and HCC progression and extended survival [51]. Itraconazole also improved the therapeutic effect of sorafenib, a multikinase inhibitor employed in HCC, in male rats [52]. The effect of itraconazole in female rodents has not been investigated to our knowledge. Women showed poorer absorption and higher metabolism of this drug, resulting in lower exposure [53]. Higher doses of the drug appear to be required for female patients.

Despite the fact that sex differences in HCC development risk are widely acknowledged, the prognosis of HCC between the sexes is still debatable. In a nationwide survey of 4649 HCC cases conducted in Japan, male sex was found to be an independent risk factor for a worse prognosis [54]. Female sex was an independent predictor of improved survival in an Italian survey of 600 untreated HCC cases [55]. Other studies, though, did not show a sex difference in the prognosis of HCC [56,57]. In a large study of HCC patients, overall survival was similar in men and women [17], consistent with our study in which overall survival, relapse-free survival, and metastasis-free survival did not differ by sex. The five-year survival rate for males in Germany is 18%, which is higher than the 14% rate for females [15], indicating that the survival rate of our HCC cohort does not correspond to that of all German cases. This may be due to the comparatively low number of female patients enrolled in our study.

A significant number of HCC patients suffer from chronic liver disease and fibrosis, and liver-specific downregulation of NPC1 in female mice caused inflammation and fibrosis [58]. NPC1 protein was increased in the livers of patients with cholestasis, as well as in mice with toxic liver injury [59]. However, in male patients with HCC, non-tumor NPC1 protein levels were not associated with fibrosis. The number of female patients in this cohort was too small for meaningful analysis. Future studies should investigate the expression of hepatic NPC1 in cases of chronic liver injury of different etiologies in both sexes.

In terms of translational value, the findings of this study suggest that NPC1 could be used as a diagnostic biomarker for HCC. The analysis also indicates that proteins involved in cellular cholesterol transport, such as NPC1, could be therapeutic targets in both sexes. Studies evaluating the effect of itraconazole on HCC, which take into account the lower drug exposure in females, are warranted [53]. Studies concerning HCC progression, the HCC proteome, and the efficacy of different drugs and therapies should be conducted in both sexes routinely.

This analysis has limitations. For about 60% of the patients, the cause of their liver disease was unknown. Another limitation is the descriptive nature of this retrospective study, which used a pathologist’s visual scoring of staining intensity to quantify the NPC1 protein. The female cohort was small, and these observations have to be confirmed in larger groups. The strength of our study lies in the large number of patients included, all of whom had their disease states well documented. This allowed us to conduct a sex-specific analysis of NPC1 protein levels in HCC tissue samples.

## 5. Conclusions

This study shows that NPC1 protein is highly induced in HCC tissue from both male and female patients. NPC1 in tumors was associated with T stage, AFP, and UICC scores in both sexes. NPC1 correlated with tumor inflammation and tumor size in women and with survival in men, showing sex-specific associations of NPC1 in HCC, the pathophysiological role of which has to be clarified.

## Figures and Tables

**Table 1:** T stage, lymph node invasion, grading, tumor size, UICC stage, survival, intratumoral inflammation, fibrosis stage, age, alpha-fetoprotein, and NPC1 protein levels of male and female patients, of whom non-tumor (NT) or tumor tissue (TT) has been obtained. Median values, minimum values, and maximum values are given in the table. ** *p* < 0.01. Superscript numbers refer to the number of females/males for whom alpha-fetoprotein was documented. In the non-HCC group, alpha-fetoprotein was documented for only a few patients and is not listed in the table (n.l.).

	Males NT	Females NT	Males TT	Females TT
Patients	41	7	264	59
T stage	3 (1–4)	3 (1–4)	3 (1–4)	3 (1–4)
Lymph node invasion	0 (0–0)	0 (0–0)	0 (0–1)	0 (0–1)
Grading	2 (1–3)	2 (1–3)	2 (1–3)	2 (1–3)
Tumor size (cm)	3.4 (0.8–18.0)	5.1 (1.5–13.7)	4.9 (0.5–25.0) **	6.9 (0.7–24.0) **
UICC score	2 (1–4)	2 (1–4)	2 (1–4)	2 (1–4)
Overall survival years	3 (0–21)	2 (1–18)	4 (0–28)	4 (1–22)
Metastasis-free years	2 (0–21)	2 (1–18)	3 (0–28)	3 (1–20)
Recurrence-free years	2 (0–21)	2 (1–18)	2 (0–20)	3 (1–22)
Intratumoral inflammation	1 (0–2)	1 (0–1)	1 (0–3)	1 (0–3)
Fibrosis grade	6 (0–6)	3 (0–6)	5 (0–6)	4 (0–6)
Age (years)	63 (38–84)	52 (35–85)	66 (10–85)	65 (25–83)
Alpha-fetoprotein (ng/mL)	n.l.	n.l.	14 (1–17 × 10^4^)^137^	73 (1–1 × 10^4^)^38^
NPC1 Protein	40 (10–180)	60 (10–80)	120 (5–300)	140 (5–300)

## Data Availability

The data presented in this study is contained within the article.
